# A system for studying mechanisms of neuromuscular junction development and maintenance

**DOI:** 10.1242/dev.130278

**Published:** 2016-07-01

**Authors:** Valérie Vilmont, Bruno Cadot, Gilles Ouanounou, Edgar R. Gomes

**Affiliations:** 1 Myology Research Center, UM76-INSERM U974-CNRS FRE 3617 Sorbonne Universités, UPMC Université Paris 06, Paris, France; 2 FRE CNRS 3693 (U.N.I.C), Unité de Neuroscience, Information et Complexité CNRS, Bât. 33, 1 Ave de la Terasse, Gif sur Yvette 91198, France; 3 Instituto de Medicina Molecular, Faculdade de Medicina da Universidade de Lisboa, Lisboa, Portugal

**Keywords:** Co-culture, Differentiation, Myofiber, NMJ

## Abstract

The neuromuscular junction (NMJ), a cellular synapse between a motor neuron and a skeletal muscle fiber, enables the translation of chemical cues into physical activity. The development of this special structure has been subject to numerous investigations, but its complexity renders *in vivo* studies particularly difficult to perform. *In vitro* modeling of the neuromuscular junction represents a powerful tool to delineate fully the fine tuning of events that lead to subcellular specialization at the pre-synaptic and post-synaptic sites. Here, we describe a novel heterologous co-culture *in vitro* method using rat spinal cord explants with dorsal root ganglia and murine primary myoblasts to study neuromuscular junctions. This system allows the formation and long-term survival of highly differentiated myofibers, motor neurons, supporting glial cells and functional neuromuscular junctions with post-synaptic specialization. Therefore, fundamental aspects of NMJ formation and maintenance can be studied using the described system, which can be adapted to model multiple NMJ-associated disorders.

## Abstract (precis)

**Summary:** Co-culture of rodent spinal cord explants and primary myoblasts provides an *in vitro* system to study fundamental aspects of neuromuscular junction formation, maintenance and dysfunction.

## INTRODUCTION

The ongoing development of new experimental approaches has proved to be useful for modeling a number of adverse health conditions, including neuromuscular diseases (Chew et al., 2015; Lenzi et al., 2015; Sandoe and Eggan, 2013; Tu et al., 1996). Neuromuscular pathologies encompass a wide range of subgroups, including (1) myopathies such as Duchenne's and Becker's muscular dystrophies (Flanigan, 2014); (2) motor neuron diseases (MNDs) such as amyotrophic lateral sclerosis (ALS), progressive bulbar palsy, pseudobulbar palsy and spinal muscular atrophy (SMA) (de Boer et al., 2014; Edens et al., 2015; Karam et al., 2010; Tu et al., 1996); and (3) auto-immune neuromuscular diseases such as myasthenia gravis and Lambert–Eaton myastenic syndrome (Ha and Richman, 2015; Lang et al., 2003). Researchers have set up different animal models and cell lines (Chen et al., 2014; Corti et al., 2012; Lenzi et al., 2015) with the hope of recapitulating some features of neuromuscular diseases and understanding the triggers of one of their common hallmarks: the disruption of the neuromuscular junction (NMJ). The NMJ is one of the most studied synapses. It is formed of three key elements: the lower motor neuron (the pre-synaptic compartment), the skeletal muscle (the post-synaptic compartment) and the Schwann cell (Sanes and Lichtman, 1999). The NMJ is formed in a step-wise manner following a series of cues involving these three cellular components and its role is basically to ensure the skeletal muscle functionality. Following an action potential down the motor neuron axon, synaptic vesicles will fuse with the membrane at the half-terminal of the axon releasing neurotransmitters into the synaptic cleft. The post-synaptic membrane of the muscle fiber is specialized to respond efficiently to the neurotransmitter release and will convert the chemical signal to a mechanical signal in the form of muscular contraction (Das et al., 2010). In some cases, the dialog between these cellular components is compromised and leads to instability of the NMJ and in worst cases, like in ALS and SMA, eventually axon retraction and muscle atrophy.

In order to study NMJ physiology and pathology, different *in vivo* systems are used, such as mouse diaphragm or *Drosophila* abdominal segments (Packard et al., 2002; Perez-Garcia and Burden, 2012). However, these systems do not allow observation and manipulation over long periods of time in live NMJ. Therefore, understanding the development of the NMJ often needs transgenic organisms, generation of which is time consuming and sometimes impossible. To overcome these problems, different *in vitro* co-culture systems have been set up in which motor neuron and skeletal muscle are grown together in order to recapitulate the formation and eventual disruption of the NMJ. To date, co-culture methods established from various species have been described, including mouse (Morimoto et al., 2013; Zahavi et al., 2015), rat (Das et al., 2010; Southam et al., 2013), *Xenopus* (Lu et al., 1996; Peng et al., 2003) and chick (Frank and Fischbach, 1979), and also heterologous co-cultures built from motor neuron and muscle cells obtained from different species, such as rat-human (Askanas et al., 1987), mouse-human (Son et al., 2011) and mouse-chick (Soundararajan et al., 2007). However, these co-culture methods resulted in the formation of immature myofibers (thin muscle fiber, with centrally localized nuclei and no transversal triads) with immature sarcomeric structures (Das et al., 2007, 2009; Southam et al., 2013). Moreover, previous models did not take advantage of their co-culture system to analyze other post-synaptic structures such as the formation of muscle-specific tyrosine kinase (MuSK) and Rapsyn (also known as Rapsn) clusters which are formed as agrin-induced signaling sparks off and which are essential to the formation of acetylcholine receptor (AChR) clusters. Here, we describe a new functional co-culture system in which muscle fibers from primary murine myoblasts are brought to advanced differentiation and form highly matured NMJs with motor neurons derived from rat spinal cord. The muscle fibers show hallmarks of mature skeletal muscle fiber: peripheral nuclei, transversal triads, myofibrils and organization into three-dimensional bundles performing synchronized contraction. Furthermore, the NMJ showed pretzel-like morphology reminiscent of *in vivo* synapses. We used this co-culture model to investigate the formation of the post-synaptic apparatus beyond the clustering of AChRs and we investigated the role of motor neuron firing on muscle development and differentiation. We found that AChRs form clusters at motor neuron-muscle contacts, that the post- and pre-synapses show hallmarks of maturation and that these NMJs are functionally active.

## RESULTS

### Development of a heterologous co-culture system

We have previously described a method for obtaining highly differentiated myofibers *in vitro*, which is potentially useful for studying myoblast fusion, nuclear movement, myofiber differentiation and formation of agrin-induced AChR clusters (Falcone et al., 2014). However, this method is not suitable for studying formation of NMJs and the post-synaptic apparatus as well as mechanisms of denervation-dependent muscle atrophy, due to the lack of two of the basic cell types forming the NMJ: neurons and Schwann cells. We therefore developed an easy co-culture system that allowed us to obtain highly differentiated myofibers in a more physiological context, i.e. innervated by neurons.

**Fig. 1.**
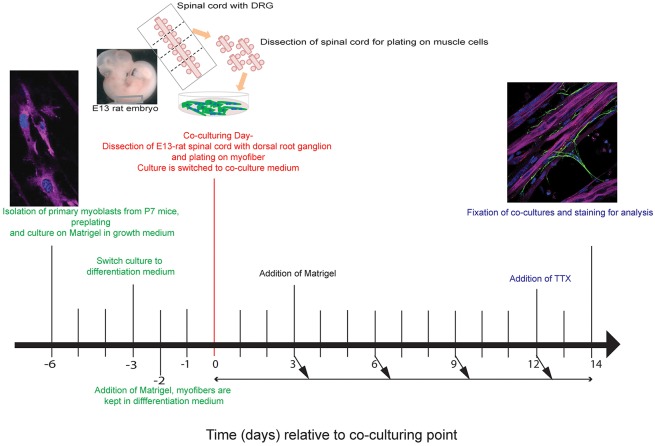
**Timeline for spinal cord explant and murine myofibers co-culture.** Days are expressed relative to day of spinal cord explant plating on myotubes. Fixation of co-culture was performed at Day 14 for staining purposes but co-cultures may be maintained for up to 4 weeks with regular medium changes.

Myoblasts were isolated from postnatal day (P) 7 mouse pups and plated on Matrigel-coated coverslips (Fig. 1, Day −6). Matrigel is rich in extracellular matrix proteins important for muscle differentiation (Falcone et al., 2014). Moreover, Matrigel represents a very suitable biomaterial for our co-culture given the presence of laminin in its components (Kleinman et al., 1982), an important element in presynaptic differentiation and organization of NMJ active zones (Nishimune, 2012; Sanes and Hall, 1979). In addition, Matrigel provided a three-dimensional matrix for our cells to grow and differentiate. Myofibers were then switched to differentiation (Fig. 1, Day −3). Twenty-four hours later (Fig. 1, Day −2), another layer of Matrigel was added to the cells to cover the differentiating muscle fibers (Fig. 1). Forty-eight hours later (Fig.1, Day 0), whole transverse sections of the spinal cord with attached dorsal root ganglia (DRGs) without removal of the ventral part, i.e. the ventral horn, where efferent nerves are believed to emanate (Blits et al., 2004), were plated on myotubes (Fig. 1). Medium was also supplemented with growth factors [brain-derived neurotrophic factor (BDNF), ciliary neurotrophic factor (CNTF) and glial cell-derived neurotrophic factor (GDNF)] involved in the maturation of NMJs (Peng et al., 2003; Sakuma and Yamaguchi, 2011; Tuttle and Matthew, 1995; Zahavi et al., 2015). After 12 days, myofibers showed high contractile activity; in order to reduce the possibility of detachment of the neuron-muscle structures from the dish, we used tetrodotoxin 1 µM (TTX). This treatment not only reduced myofiber contractions, but also enhanced formation of neuromuscular junctions. Time-course analysis showed that at 14 days after starting the co-culture, fully mature myofibers and functional NMJs were present. In addition, we found that the co-culture could be kept over longer time periods (at least 30 days) without affecting the viability of the neurons or myofibers, suggesting that we were able to generate a system that is stabilized by a complex dialog between different cell types.

### Characterization of co-culture morphology

**Fig. 2.**
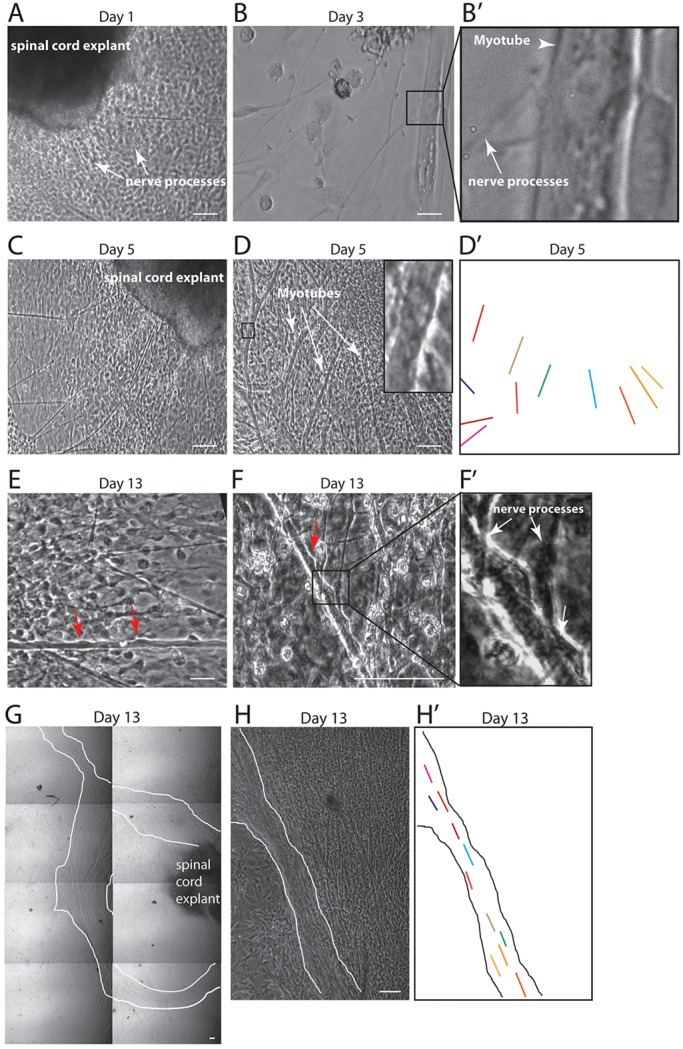
**Morphological characterization of co-culture.** (A) Day 1. Spinal cord explant showing extensions of nerve processes (arrows) on top of primary myoblasts. (B,B′) Day 3. Nerve processes (arrow) forming contact with myotubes (arrowhead). Boxed area is magnified in B′. (C) Day 5. Nerve processes are longer and extended over large distances away from the spinal cord explant. (D,D′) Day 5. Myotubes are still immature. They do not show peripheral nuclei (inset shows magnification of boxed area). As shown by colored lines highlighting outlines of myofibers in D′, the myofibers do not show alignment. (E) Day 13. Myofibers with peripheral nuclei, a hallmark of differentiation (red arrows). (F,F′) Day 13. Myofibers show peripheral nuclei (red arrow) and multiple contacts with nerve processes (white arrows). Boxed area is magnified in F′. (G) Day 13. Myotubes form bundles around the spinal cord explant. Metamorph software was used to acquire adjacent images with a 4× objective in order to cover a surface of 1.5 cm in height and 1 cm in width. (H,H′) Day 13. Myofibers show bundling and their regular alignment is shown in H′. Aligned myofibers bundle is outlined. Scale bars: 100 µm (A-H).

Study of the morphology of the co-cultures over time showed that at Day 1 post-culturing, the explants already projected nerve processes (Fig. 2A). If, by Day 1, explants had not shown any nerve processes, the explants will certainly not adhere to the muscle and were removed. At Day 3, neuron-muscle contacts could already be spotted in the cultures (Fig. 2B,B′). At Day 5, the nerve processes had extended very widely (Fig. 2C) and cell types other than neurons were present in the cultures (Fig. 2C), but muscle fibers still exhibited an immature differentiation status (Fig. 2D) and no alignment, as shown by the colored outlines of myofibers (Fig. 2D′). At Day 13, however, myofibers showed hallmarks of differentiation, such as peripheral nuclei (Fig. 2E,F, red arrows), as well as numerous contacts with neurons (Fig. 2F,F′). Movement of myonuclei to the periphery, although not yet completely understood, has been described as an important characteristic of muscle differentiation, and failure of re-positioning of myonuclei from center to periphery is pervasive in centromyonuclear myopathies such as X-linked myotubular myopathy (Folker and Baylies, 2013). Although at Day 5 muscle cells showed random positioning in the culture, at Day 13, interestingly, the muscle fibers showed alignment around the explant (Fig. 2G,H) reminiscent of the formation of bundling of multiple myofibers *in vivo*. In Fig. 2H′, outlines of myofibers allow the regular myofiber alignment to be distinguished. Altogether, these results show that our protocol allowed for the formation of axonal processes, muscle-nerve contacts and features of muscle differentiation over a short time period.

### Characterization of co-culture cell components: neuronal populations

**Fig. 3.**
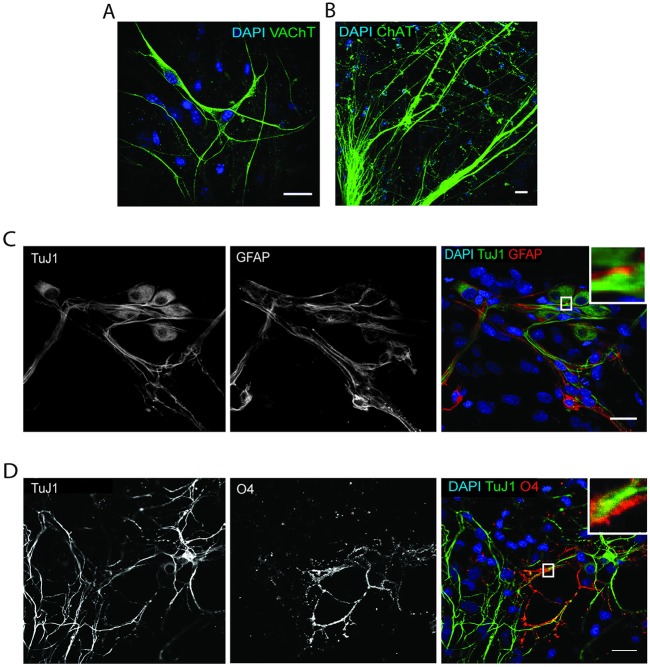
**Characterization of neuronal populations at Day 14.** (A) Representative images of co-culture stained for VaChT (green) and DAPI (blue). (B) Representative image of co-culture stained for ChAT (green) and DAPI (blue). (C) Representative image of co-culture stained for β-III tubulin (TuJ1; green), GFAP (red) and DAPI (blue). (D) Representative image of co-culture stained for β-III tubulin (TuJ1; green), O4 (red) and DAPI (blue). Insets show magnifications of boxed areas. Scale bars: 20 µm.

We investigated the presence of different cell types by first looking at the presence of cholinergic motor neurons, known to form the NMJ upon contact with the muscle (Feng and Dai, 1990). To this end, we marked the co-cultures with vesicular acetylcholine transporter (VaChT; also known as Slc18a3) and choline acetyltransferase and (ChAT) antibodies (Schäfer et al., 1998) and found that the co-cultures contain many cholinergic neurons (Fig. 3A,B).

The presence of cells other than neuron and muscle has not been previously described in methods of co-cultures. We were particularly interested in the presence of glial cells, especially Schwann cells, given their importance in the formation and nurturing of the NMJ (Sanes and Lichtman, 1999; Witzemann, 2006). To this end, we used the following macroglial cell markers: glial fibrillary acidic protein (GFAP), which marks non-myelinating Schwann cells (Jessen and Mirsky, 2005; Jessen et al., 1990; Kegler et al., 2015), and the oligodendrocytic marker O4, which marks oligodendrocytes (Gorris et al., 2015). Schwann cells are indispensable for nerve capping and production of the myelin sheath, which speeds conduction of the action potential along axons and insulates axons to avoid energy loss (Jessen and Mirsky, 2005; Sanes and Lichtman, 1999). Oligodendrocytes are the central nervous system (CNS) equivalent of Schwann cells. We found both Schwann cells and oligodendrocytes, the latter probably emanating from the ventral horn (Wada et al., 2000), in our co-cultures, at Day 14 (Fig. 3C,D). Interestingly, both cells types showed contact points with neurons, suggesting capping of axons as has been described *in vivo* (Fig. 3D, inset). Overall, the presence of these different neuronal cell types, with a localization resembling observations *in vivo*, supports the notion that our system enables the development of efficient motor neurons.

### Characterization of co-culture cell components: myofibers

**Fig. 4.**
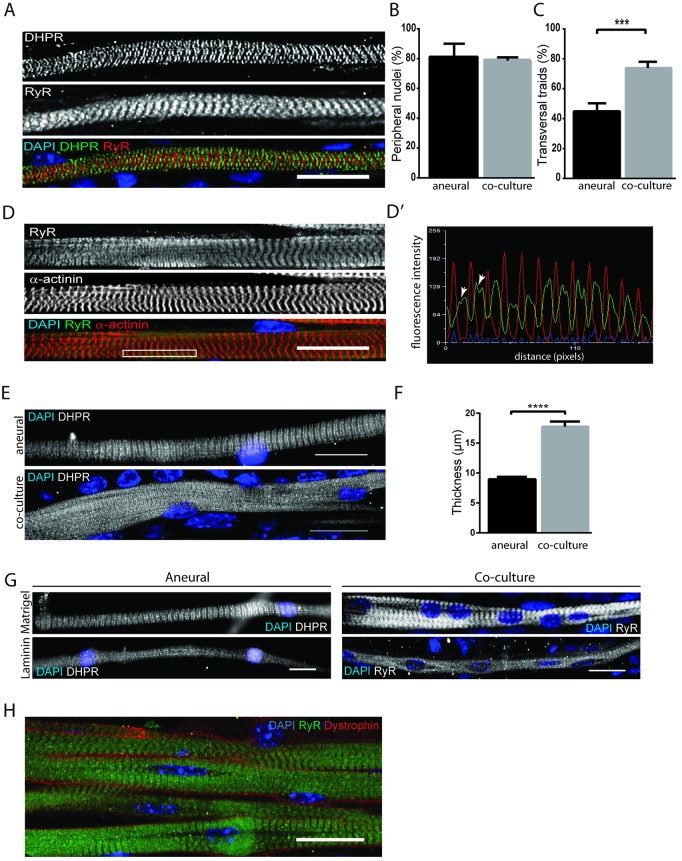
**Characterization of myofibers at Day 14.** (A) Representative *z*-projection of differentiated myofiber stained for DHPR (green), RyR (red) and DAPI (blue). (B) Quantification of myofibers with peripheral nuclei in aneural vs neural conditions. Error bars indicate s.e.m.; 67 myofibers in aneural and 87 myofibers in co-culture have been counted in three independent experiments. *P*-values from Welch's *t*-test. (C) Quantification of myofibers with transversal triads in aneural versus neural conditions. Error bars indicate s.e.m.; 67 myofibers in aneural and 87 myofibers in co-culture have been counted in three independent experiments. ***P<0.001 (Welch's *t*-test). (D) Representative *z*-projection of differentiated myofiber stained for RyR (green), α-actinin (red) and DAPI (blue). (D′) Line scan of boxed region in D showing average intensity of RyR compared with α-actinin. Arrowheads indicate doublets. (E) Representative images of differentiated myofibers in aneural and co-culture conditions. (F) Quantification of myofiber thickness in aneural versus co-culture conditions. Error bars indicate s.e.m.; 44 myofibers in aneural and 40 myofibers in co-culture have been counted in three independent experiments. ****P<0.0001 (Mann–Whitney test). (G) Representative image of myofibers, stained for DHPR or Ryr (gray) and DAPI (blue), grown on Matrigel versus laminin in aneural versus neural conditions. (H) Representative *z*-projection image of myofiber bundle stained for RyR (green), dystrophin (red) and DAPI (blue) in co-culture conditions. Scale bars: 20 µm.

We previously described an *in vitro* model of differentiated myofibers displaying several features indicative of maturation, including the presence of T-tubules and sarcoplasmic reticulum (SR) evenly and transversally organized (Falcone et al., 2014). These differentiated myofibers were formed in the absence of neurons, and therefore we named it an aneural system. To characterize the maturation of myofibers in our co-culture and aneural systems, we used antibodies against the dihydropyridine receptor (DHPR), a voltage-gated channel found at the T-tubule, or against ryanodine receptor (RyR), which is found at the sarcoplasmic reticulum membrane (Flucher et al., 1993, 1994). Both receptors are implicated in the excitation-contraction (EC) coupling mechanism, through the existence of triads, where one T-tubule is coupled to two terminal cisternae of the sarcoplasmic reticulum. At Day 14, myofibers in the co-culture system showed features of advanced differentiation: well-formed DHPR-positive triads and peripheral nuclei (Fig. 4A). In the co-culture system, we found that the percentage of myofibers with peripheral nuclei was similar in both systems (Fig. 4B). However, the number of fibers with triads was higher than at the endpoint of the aneural system (Day 10) (mean±s.e.m., 78.6%±2.3 versus 47%±5.6; Fig. 4C).

To characterize further the degree of muscle differentiation, we stained our co-cultures for α-actinin, an actin-binding protein found at the z-disk. α-Actinin binds actin and several other proteins, such as titin, and forms a lattice-like structure that is important for the stabilization of the muscle contractile apparatus (Sjöblom et al., 2008). Day 14 myofibers showed regular alternation between the z-disk and doublets structures marked with α-actinin and RyR, respectively, as shown on the line scan (Fig. 4D,D′; RyR doublets are marked with arrowheads).

Comparison of myofiber thickness from the co-culture and aneural systems confirmed that the presence of neurons definitely promoted the formation of thicker fibers (Fig. 4E). Myofibers from the co-culture showed a 1.8-fold increase in thickness (mean±s.e.m., 17.4 µm±0.72 versus 9.38±0.36; Fig. 4F). Of note, when Matrigel was substituted with laminin, a component of muscle basal lamina, the differentiation status of myofibers was negatively affected, suggesting that laminin alone, though intensively nourishing to myofibers, could not account for the high myofiber differentiation (Fig. 4G). Furthermore, as we described in Fig. 2H, myofibers showed bundling similar to *in vivo* fasiculus, a feature important for increased contractile strength (Fig. 4H). Taken together, these data demonstrate that our protocol allows for robust differentiation of thick myofibers with peripheral nuclei and transversal triads and, importantly, that the presence of neurons is a determining factor in the differentiation process of myofibers.

### Formation of NMJs

**Fig. 5.**
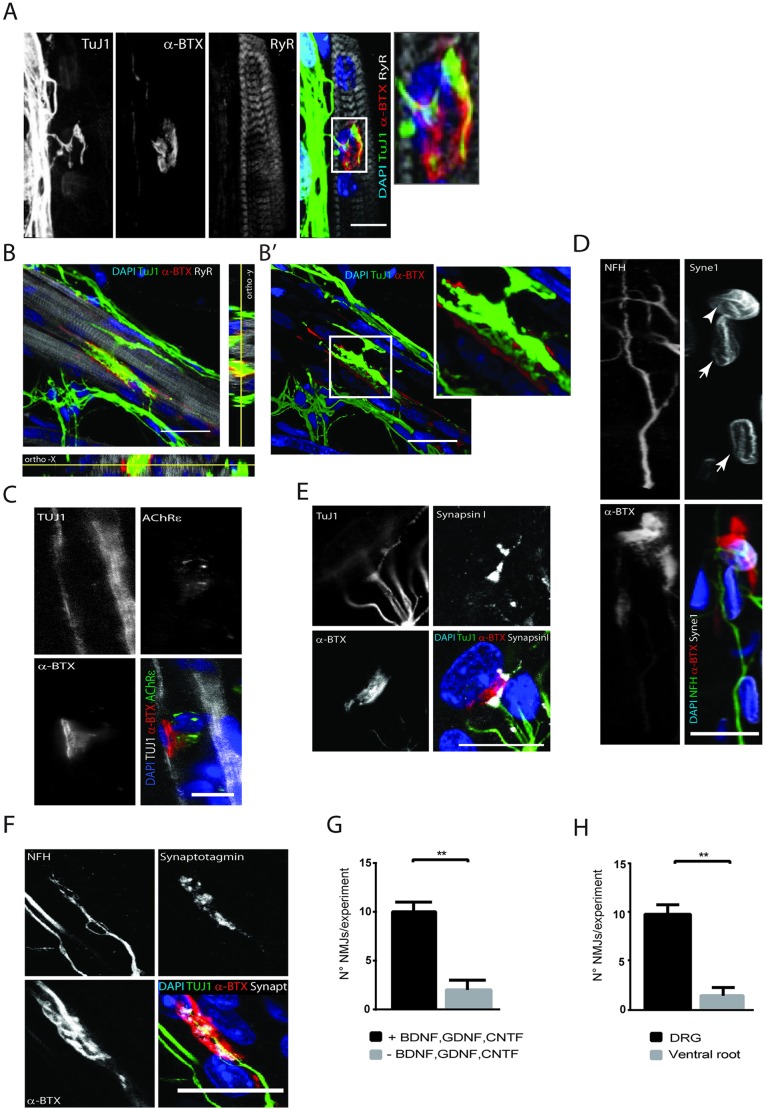
**Characterization of neuromuscular junction at Day 14.** (A) Representative image of co-culture stained for TuJ1 (green), AChRs (α-BTX) (red), RyR (gray) and DAPI (blue). Colocalization of AChR clusters with nerve terminal (boxed area) is magnified. (B) Representative *z*-projection of a co-culture showing NMJ complexity. Orthogonal view in *x*-axis and *y*-axis confirm colocalization of AChR nerve endings. (B′) 3D reconstruction image of the NMJ shown in B without the myofiber, showing interaction between AChR clusters and nerve endings (boxed area magnified in inset). (C) *In situ* hybridization detects AChRε at NMJ. The presence of AChRε is detected with a red fluorescent probe (marked in green in the figure), presynaptic terminal stained for TuJ1 (gray), post-synaptic terminal for α-BTX (red) and DAPI (blue). (D) Representative image of presynaptic terminal stained for NFH (green), α-BTX (red), Syne-1 (gray) and DAPI (blue). Extrasynaptic nuclei of same fiber indicated by the arrows show decreased Syne-1 expression compared with the synaptic nucleus indicated by the arrowhead. (E) Representative image of co-culture stained for TuJ1 (green), α-BTX (red), synapsin I (gray) and DAPI (blue). (F) Representative image of presynaptic terminal stained for TuJ1 (green), α-BTX (red), synaptotagmin (gray) and DAPI (blue). (G) Quantification of NMJs with or without addition of BDNF, GDNF and CNTF. Error bars indicate s.e.m.; three independent experiments. **P<0.01 (Welch's *t*-test). (H) Quantification of NMJs in whole spinal cord explants versus ventral root explants. Error bars indicate s.e.m.; three independent experiments. **P<0.01 (Welch's *t*-test). Scale bars: 10 µm (A,E); 20 µm (B-D,F).

NMJ are characterized on the postsynaptic membrane (the myofiber plasma membrane) by the presence of highly clustered acetylcholine receptors, detected by α-bungarotoxin (Sanes and Lichtman, 1999; Wu et al., 2010). In several previous protocols that described NMJ formation, the researchers have presented NMJs as random colocalization points between the neuron and the myofiber with dotty and/or unstructured AChR clusters (Das et al., 2007; Southam et al., 2013), usually formed before any innervation. However, *in vivo*, neuron-muscle fortuitous colocalization alone is not a satisfactory feature (Thomson et al., 2012). Early characterization of NMJs has shown that, as opposed to small unspecialized aneural prepatterned AChR clusters, axons end perfectly at the post-synaptic terminal and overlap the terminal where AChR clusters will mature to form complex structures (Englander and Rubin, 1987; Sanes and Lichtman, 1999). Remarkably, in our co-culture system at Day 14, axons extended to innervate myofibers above complex AChR clusters with a similar morphology to NMJs found *in vivo*, i.e. a characteristic pretzel shape with typical internal perforations, an indication of low-density AChR spots (Fig. 5A). We also found few unstructured AChR clusters at un-innervated myofibers (Fig. S1). Given that three layers of Matrigel were used in order to create an elaborate 3D matrix, we analyzed orthoslices of NMJ stacks and found elaborate capping of AChR clusters by nerve terminals with the presence of synaptic nuclei below. The complexity of NMJ structures (Fig. 5B) was revealed using 3D reconstruction whereby the myofiber channel was removed to take full account of the capping of the AChR clusters by the neuron (Fig. 5B′ and inset).

The NMJ is also characterized by accumulation in the myofiber of specific mRNAs, such as the AChR epsilon subunit mRNA (Gu and Hall, 1988; Merlie and Sanes, 1985), and accumulation of Syne1 protein by subsynaptic nuclei at the nuclear envelope (Apel et al., 2000). We found that AChR epsilon subunit mRNA accumulates at the NMJ in our co-culture system (Fig. 5C). Furthermore, subsynaptic nuclei also accumulate more Syne1 at the nuclear envelope (Fig. 5D). Additionally, we investigated whether the NMJ bore active zones at presynaptic terminals. To this end, we stained for synapsin I, a synaptic vesicle protein; synaptotagmin, a calcium sensor also important for the vesicle docking process; and bassoon, a scaffolding protein believed to guide the synaptic vesicles to the active zones (Fornasiero et al., 2012; Reist et al., 1998; Südhof, 2012; Willig et al., 2006; Zhai et al., 2001; Ziv and Garner, 2004). We found that all these proteins were enriched specifically at the pre-synaptic terminals of the NMJ (Fig. 5E,F; Fig. S3).

Finally, we found that the growth factors that were supplemented at Day 0 (BDNF, CNTF and GDNF) were crucial for the formation of the NMJ as the number of NMJs was dramatically reduced in the absence of these growth factors (mean±s.e.m., 10±1 versus 2±1; Fig. 5G). We tested co-cultures with transverse sections of ventral root stripped of meninges and DRGs under the same conditions with growth factors and found, in accordance with other reports, that the number of NMJs was drastically reduced (Guettier-Sigrist et al., 1998; Kobayashi et al., 1987) (Fig. 5H).

**Fig. 6.**
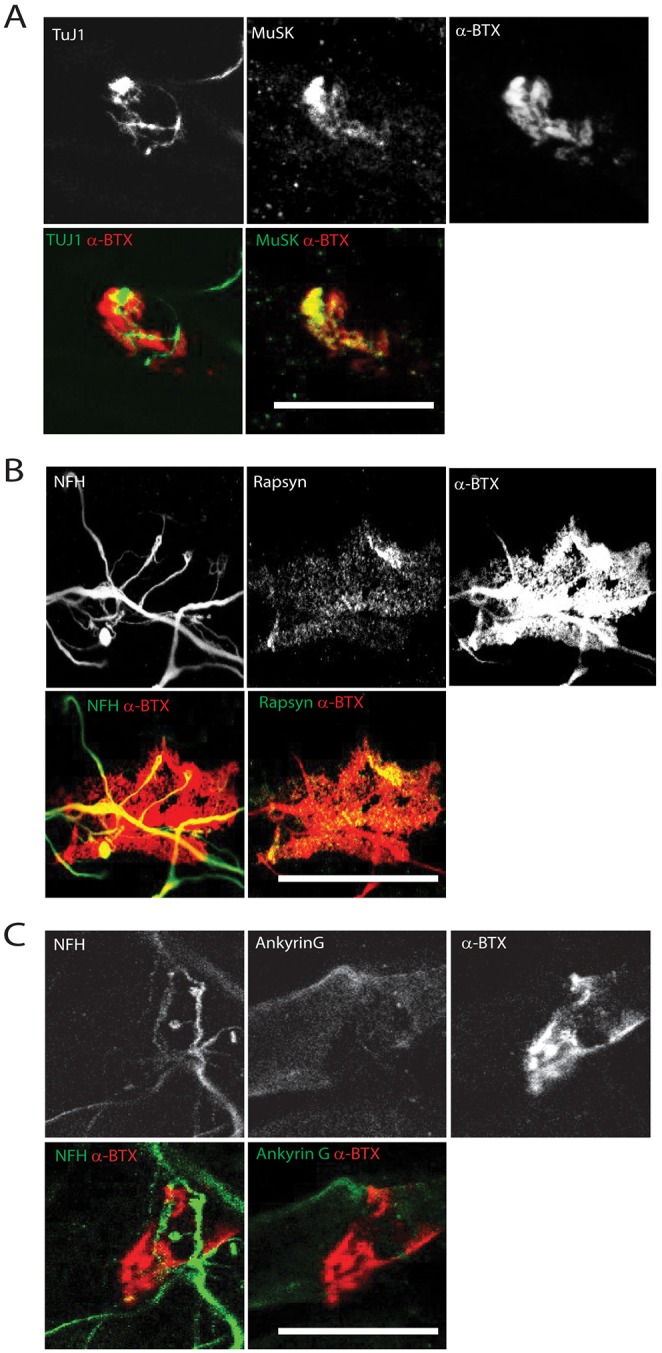
**Characterization of post-synaptic specialization at Day 14.** (A) Representative *z*-projection of differentiated myofibers stained for α-BTX (red) and TuJ1 or MuSK (green). (B) Representative *z*-projection of differentiated myofibers stained for α-BTX (red) and NFH or Rapsyn (green). (C) Representative image of differentiated myofibers stained for α-BTX (red) and NFH or ankyrin G (green). Scale bars: 20 µm.

Following our data showing that our co-culture system leads to the formation of highly differentiated NMJs, we sought to assess whether our system could allow us to study specifically the regionalization of different post-synaptic elements. To this end, we investigated the spatial organization of different components: MuSK, which is found at the membrane of primary gutters like AChR, and Rapsyn, which is essential for the formation and maintenance of the NMJ (Wu et al., 2010). Upon neuronal agrin secretion, several signaling pathways are activated at the post-synapse (Luo et al., 2003). Among these pathways is the dimerization and self-activation of MuSK via phosphorylation (Hopf and Hoch, 1998; Kim et al., 2008; Zhang et al., 2008). Subsequently, interactions between the kinase and other synaptic proteins will increase and trigger postsynaptic differentiation. These events are crucial to AChR clustering. Agrin also induces association of AChRs with Rapsyn, an important stabilizer of AChR clusters (Apel et al., 1997; Chen et al., 2007). We found that MuSK and Rapsyn colocalize with AChR clusters, as expected (Fig. 6A,B). Interestingly, MuSK could be found at the muscle plasma membrane, in line with its receptor tyrosine kinase functions (Fig. 6A). We also investigated the localization of ankyrin G (also known as ankyrin 3), which is known to be spatially segregated from AChR at the NMJ in early development and then found at secondary junctional folds later in development (Trinidad et al., 2000; Wood and Slater, 1998). We found that ankyrin G was clustered in close proximity to the NMJ and was found at the muscle membrane but not actually at the NMJ (Fig. 6C), suggesting that at this time of development of the system, the protein localization has not reached a fully mature status as previously reported *in vivo* (Bailey et al., 2003), further supporting this method for the study of different phases of NMJ maturation.

Overall, these data demonstrate the uniqueness and robustness of our system for studying NMJ formation and post-synaptic development.

### NMJ functionality

**Fig. 7.**
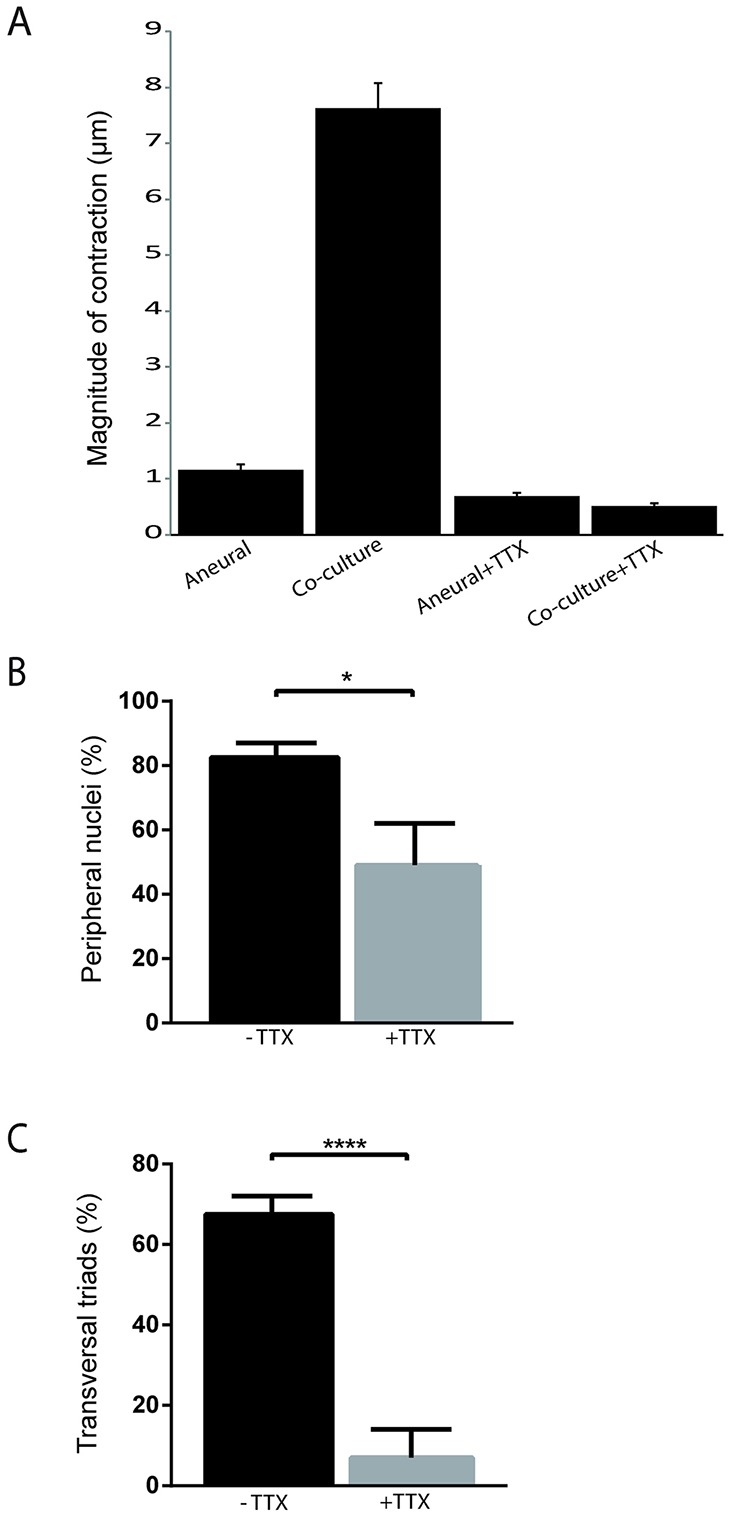
**Differences between muscle contraction in aneural versus co-culture conditions.** (A) Average amplitude of contraction observed over 80 s (equal to 160 frames) has been calculated for each condition (± TTX). Error bars indicate s.e.m; *n*=160. (B) Quantification of myofibers with peripheral nuclei with or without addition of TTX at early time points (day 4 until day 14). Error bars indicate s.e.m.; three independent experiments. *P<0.05 (Welch's *t*-test). (C) Quantification of myofibers with triads with or without addition of TTX at early time points (day 4 until day 14). Error bars indicate s.e.m.; three independent experiments. ****P<0.0001 (Welch's *t*-test).

The structural characterization of our co-culture showed the presence of different basal elements organized specifically to allow for a functional neuromuscular system, from the motor command to the myofiber contraction. Both innervated myofibers and aneural myofibers showed contractions (Fig. 7A; Fig. S2A; Movies 1 and 2). The contractile activity by itself showed the functionality of the contractile apparatus and of the excitation-contraction coupling. Indeed, 1 µM tetrodotoxin (TTX), a selective blocker of voltage-gated sodium channels, abrogated contractile activity in both neural (Fig. 7A; Fig. S2B; Movie 1) and aneural (Fig. 7A; Fig. S2C; Movie 2) cultures. This suggests that sodium action potentials activate normally the DHPR voltage sensors at the level of the T-tubules, and subsequently the calcium release from the terminal cisternae of the sarcoplasmic reticulum and the contraction. At first glance, the contractile activity, however, does not allow conclusions to be made regarding the efficiency of the synaptic transmission, because myofibers in aneural culture spontaneously twitch (Fig. 7A; Fig. S2A; Movie 2). Nonetheless, a more detailed analysis showed that the temporal pattern of the contractile activity changed in the presence of explants after putative NMJs are formed. Although aneural cultures exhibited rhythmic twitching activity in some individual myofibers, contractile activity in co-cultures, in proximity to the explants, was arrhythmic, with longer contraction events, and was observed in a higher number of myofibers than in aneural cultures (Fig. 7A; Fig. S2A; Movie 1). In addition, myofibers that have bundled together tend to contract simultaneously, as would be the case in a motor unit, i.e. a set of myofibers innervated by the same motor neuron. As electrical activity is known to participate in myofiber development (Dutton et al., 1993), we tested the effect of TTX on muscle differentiation. When TTX was applied early to the co-culture (Day 4 to Day 14), differentiation of myofibers was severely affected and resulted in a decreased number of peripheral nuclei (mean±s.e.m., 82.5%±5.5 versus 49%±13; Fig. 7B) and transversal triads (mean±s.e.m., 67.5%±4.5 versus 7%±7; Fig. 7C).

**Fig. 8.**
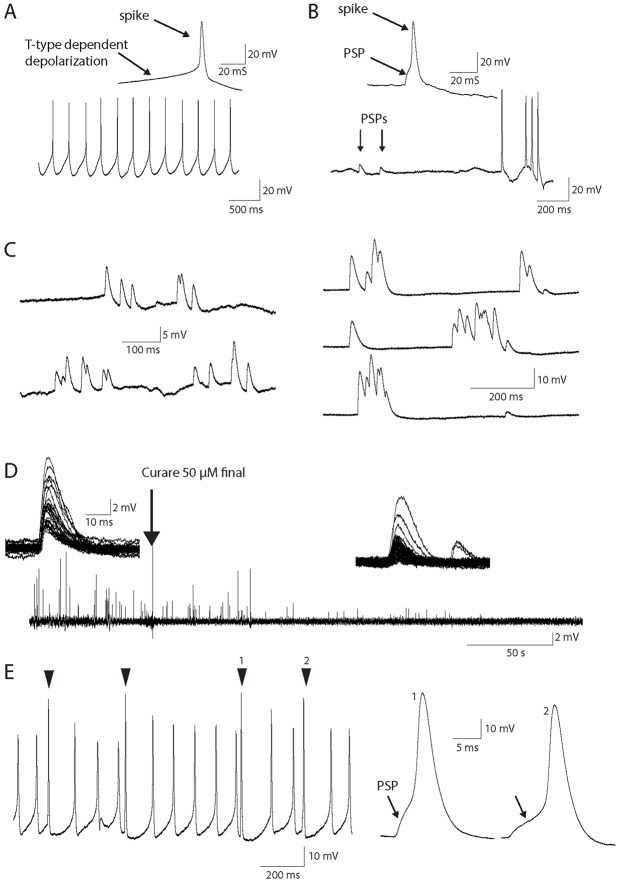
**Intracellular recordings of myofiber membrane potential.** (A) Lower trace: representative recording of the spontaneous electrical activity found in some myofibers, independently of innervation. Upper trace: enlarged time scale showing the moments preceding the spike. Depolarization to the spike threshold is due to activation of a T-type calcium current (Sciancalepore et al., 2005). (B) Lower trace: membrane potential recording in an innervated and non-spontaneously active myofiber. Arrows mark the occurrence of two sub-threshold postsynaptic potentials (PSPs). Upper trace: enlarged time scale showing the moments preceding the spike and pointing out the PSP triggering the spike. (C) PSPs in two different innervated myofibers (left and right traces, respectively). (D) PSP blockade with 50 µM curare puffed in the recording chamber. Upper traces show the individual PSPs before and after curare application. Sensitivity to curare confirmed the nicotinic nature of these PSPs. (E) Example of a recording showing a combination of spontaneous firing with synaptic-induced spikes. Arrowheads mark the spikes induced by synaptic events. Right traces: enlarged time scale for two spikes induced by PSPs.

In order to test whether synaptic transmission is functional and responsible for the arrhythmic activity in our co-culture, we performed electrophysiological recordings. Electrophysiological studies were performed in conditions of diluted Matrigel. These conditions lead to decreased myofiber differentiation, but we postulated that if the NMJs were still functional in these conditions, they would demonstrate functionality in the optimum co-culture conditions. In twitching myofibers, membrane potential recordings showed the regular muscle firing already described in aneural mice cultures (Sciancalepore et al., 2005) (Fig. 8A). This spontaneous firing is independent of synaptic activity, and is rather due to the activation of a T-type calcium current in the sub-threshold range of the membrane potential, which raises the membrane potential to the action-potential threshold (Fig. 8A, inset). This regular firing, as well as its associated contraction activity, was insensitive to 50 µM curare, a specific nicotinic receptor antagonist. In irregular-contracting myofibers, the electrical activity consisted of non-rhythmic sub-threshold depolarizations and action-potentials (Fig. 8B). Fig. 8C shows examples of these transient depolarizations, in the absence of spikes, showing their sub-threshold amplitudes or depolarization-induced inactivation of the sodium channels. Their individual temporal pattern perfectly fitted those of nicotinic post-synaptic potentials (PSPs) as usually recorded in adult murine innervated muscles, strongly suggesting that synaptic transmission is functional in the co-cultures (Ouanounou et al., 2016). The nicotinic nature of these PSPs could finally be confirmed by blockade with curare (Fig. 8D). Curare was puffed in the recording chamber (50 µM final concentration) and the diffusion time allowed to observe the gradual blockade of the post-synaptic potentials (Fig. 8D, insets). Supra-threshold post-synaptic potentials were responsible for the irregular firing in these innervated myofibers, and were visible at an enlarged time scale in the milliseconds preceding the spikes (Fig. 8B, insets). Fig. 8E shows a recording in an innervated myofiber exhibiting both spontaneous and synaptic-induced activity. In conclusion, although the conditions had to be modified for electrophysiological recordings, the data demonstrated the functionality of the nicotinic synaptic transmission in the co-culture.

Altogether, these results provide evidence for the formation of functional NMJs between neurons and highly differentiated myofibers.

## DISCUSSION

Here, we described a heterologous co-culture system capable of inducing sturdy differentiated NMJs and myofibers *in vitro* that can be directly observed by time-lapse microscopy over the whole period of differentiation. Furthermore, the myofibers can be manipulated by transfection with siRNAs and shRNAs to downregulate protein levels, or by overexpression of plasmids encoding multiple proteins (Falcone et al., 2014). To our knowledge, such an advanced stage of NMJs has never been obtained in *in vitro* conditions; therefore, this system offers unique conditions for the study of the complex mechanisms involved in the formation of NMJs and the differentiation of myofibers, which are difficult to identify using the currently available *in vivo* systems.

This method is very practical to implement in terms of availability and abundance of material and is time-saving. One pregnant rat usually bears a mean of 13-15 embryos, providing an important amount of material for obtaining spinal cord explants, allowing for the assessment of multiple culture conditions in parallel. In the present study, functional and highly differentiated NMJs, neurons and myofibers were obtained already at 14 days (Fig. 5). Other studies had used lengthy protocols by which formation of NMJs was achieved after 20-25 days or even longer time periods, if we take into account the time needed for either prior motor neuron or myofiber differentiation (Das et al., 2010; Southam et al., 2013). In addition, in these studies, the NMJs and myofibers did not reach the same stage of differentiation as that described in this manuscript.

We used spinal cord explants with dorsal root ganglia (DRGs) as a source of neuronal populations (Askanas et al., 1987; Zuchero, 2014). In several studies, however, the spinal explant was stripped of DRGs prior to plating with muscle cells to enrich the culture in motor neurons. The reason for this is that the DRG give rise to afferent sensory neurons, which are supposed to vehicle the electrical impulse from the peripheral nerve system to the brain, rather than efferent α-motor neurons, which trigger muscle contraction (Southam et al., 2013; Zahavi et al., 2015). We tried to co-culture rat spinal cord's ventral horn (stripped of DRGs) in the same conditions as described in Fig. 1. Surprisingly, the number of NMJs was smaller than that which we could achieve in our present setting. This prompts the consideration that specific neuronal populations arising from the dorsal root, including afferent neurons, could possibly be important for the full function of the system, as suggested *in vivo* (Zhang et al., 2015). Indeed, these neurons could signal, in turn, the differential contractile status of the muscle to the α-motor neurons. This also suggests that the complete spinal cord transverse sections (with the DRGs) probably host the progenitor cells necessary to differentiate into possible NMJ feeder and sustaining cells.

Our system has the unique capacity of forming highly differentiated myofibers harboring highly differentiated endplates. However, we were particularly interested in characterizing the differentiation of muscle fibers in a neuronal context, replicating *in vivo* conditions. Much attention has been given to the development and improvement of the neuronal compartment with the emergence of embryonic stem cells (Chipman et al., 2014; Das et al., 2007; Soundararajan et al., 2007) and induced pluripotent stem cells (Bohl et al., 2015; Burkhardt et al., 2013; Egawa et al., 2012); however, the muscle counterpart has been underexploited and its potential underestimated. Previous studies failed to produce convincing data on muscle differentiation either because muscle cell lines, such as C2C12, incapable of producing highly differentiated myofibers, were used or because the muscle compartment was overlooked (Arnold et al., 2012; Chipman et al., 2014; Das et al., 2009, 2010; Umbach et al., 2012). The use of such cell lines might also preclude synaptogenesis and/or synaptic differentiation. Here, we achieved substantial differentiation of myofibers that exhibited peripheral nuclei, well-organized transversal triads, and thickness to a greater extent than what we previously reported in our aneural myofiber cultures (Falcone et al., 2014) (Fig. 4). This method provides, therefore, a relatively effective developmental environment both mechanically (via neuronal impulses) and chemically, given the multiple growth factors in the Matrigel, for muscle growth and maturation (Maffioletti et al., 2015).The effect of Matrigel constituents on muscle progenitor populations, and hence muscle growth and differentiation, has already been proven (Grefte et al., 2012) and could explain why our system results in thicker and more differentiated myofibers in comparison with other protocols not using Matrigel. Among the different Matrigel constitutents, insulin-like growth factor 1 (IGF1) is known to enhance DHPR function and expression and henceforth EC machinery, important for the conversion of chemical signaling into mechanical signaling, which is correlated with muscle development and thickness.

This system also allows long-term survival of the components. Co-cultures could be kept more than 30 days with muscle fibers still contracting. It is probable that such co-cultures could be kept for even longer time periods as it has been shown previously with dorsal root ganglion/spinal cord explants and primary myoblasts co-culture (Askanas et al., 1987). The longevity of the culture would be advantageous for studying motor neuron diseases that encompass slow deterioration of the NMJ. As such, subcellular postsynaptic changes could be followed over time, for instance, with live imaging.

With the advances made in the field of embryonic stem cells (ESCs) and induced pluripotent cells (iPSCs), we think that our system could be useful for understanding the basic conditions necessary for synaptogenesis. In fact, although, any motor neuron could form a NMJ with any muscle fiber, additional signals will direct NMJ formation in favor of the ideal partners. This can only be achieved in the presence of all required cell types. We believe that although new models derived from ESC-MNs and iPSC-MNs allow for homologous co-culture systems and also hold promise for personal-targeted therapies, these systems might lack some of the required cues that allow for the precise formation of the NMJ structures, such as junctional folds in the muscle fiber (Chipman et al., 2014; Son et al., 2011). Moreover, maintenance of the co-culture over time in these systems is fragile and still needs to be improved. The issue of compromised life-time in stem cell cultures mainly arises from the differences in cell culture media requirements. ESC-derived human motor neurons are infamously difficult to culture and can develop only in complex culture medium requiring diverse trophic factors as supplements. Over time, these culture media can be pernicious to skeletal muscle. Although these techniques should be given all due interest, the cost, time and technical skills involved are not insignificant. Here, we provide an interesting alternative technique that is more time- and cost-effective.

Because one of the major technical breakthroughs in the study of NMJ formation has been the microfluidics system allowing spatial separation of the neuronal component from the muscle component, the system we describe here can be adapted for use with microfluidic devices. We believe that such a method could allow both (1) growth of all the necessary neuronal cells (neurons and glial cells) and (2) the natural dynamics of paracrine secretion of trophic factors important for synaptogenesis to occur, as suggested in the recent work of Zahavi et al. (2015).

To summarize, our work could be a valuable tool for studying the formation and development of the NMJ. We believe that because the NMJ is a perfect spatial and chemical organization between neurons, muscle and glial cells, studying all these components together could permit a better understanding of the physiology of NMJs and the temporal defaults occurring in motor neuron diseases such as ALS, spinal bulbar palsy and SMA.

## Conclusion

Our objective was to set up a new easy and durable co-culture system that would enable the fast and robust differentiation of myofibers and the formation of functional NMJs. We made use of Matrigel to create a culture platform that allowed 3D growth of neuron-muscle structures. Our method achieved the formation of myofibers exhibiting features of advanced differentiation as well as formation of functional NMJs with post-synaptic specialization. This model could be used for pharmacokinetics and drug design purposes in NMJ defect-associated pathologies such as MNDs.

## MATERIALS AND METHODS

### Animals

All animal experiments were approved by the Animal Ethics Committee of Pierre et Marie Curie University. E13 pregnant rats were obtained from Janvier Labs, St Quentin Fallavier, France. Primary murine myoblasts were obtained from animals of either sex at P7.

### Reagents

Matrigel basement membrane matrix was purchased at Corning Life Sciences (354230). Matrigel protein concentration as obtained by the Lowry method ranged between 9.2 and 10.4 mg/ml and endotoxin as measured by Limulus amoebocyte lysate assay was <1.5 EU/ml. Collagenase, recombinant BDNF and insulin were purchased from Sigma (C9263, B3795 and I1882, respectively). Dispase II was purchased from Roche (Neutral protease, grade II, 04942078001). Chicken embryo extract was purchased at MP Biochemicals (MP Biochemicals, 2850145). Recombinant rat CNTF and recombinant rat GDNF were purchased at R&D Systems (557-NT-010 and 512-GF-010, respectively). Tetrodotoxin (TTX) was purchased at Alomone Labs (T-550). Tubocurarine was purchased at Sigma-Aldrich (T2379).

### Primary skeletal muscle culture and differentiation

Skeletal muscle culture for the aneural system has been previously described (Falcone et al., 2014). Skeletal muscle cultures for the co-culture system were performed as previously described (Falcone et al., 2014) and with the following alterations. Tibialis anterior, extensor digitorum longus, gastrocnemius and quadriceps from thigh muscles were sampled from P7 pups. Muscles were minced manually then digested in 0.5 mg/ml collagenase and 3.5 mg/ml dispase at 37°C for 1.5 h (Fig. 1, day −6). Digestion was stopped with dissection medium consisting of IMDM with Glutamax (Gibco, 31980-022), 1% penicillin/streptomycin (Gibco, 15140-122) and 10% fetal calf serum (Eurobio, CVFFCSF00-01). Cells were centrifuged at 600 rpm (72 ***g***) for 6 min and the supernatant was recovered and centrifuged at 1300 rpm (340 ***g***) for 7 min. Cell pellet was re-suspended in dissection medium and filtered on a 40 µm cell strainer (Falcon Corning, 352340) and re-suspended in dissection medium in 100 mm Petri dishes (Falcon Corning, 353025) for pre-plating. Pre-plating was carried out over 3 h. Fluorodishes suitable for immunofluorescence (3.5 mm; World Precision Instruments, FD35-100) were coated with Matrigel. After pre-plating, cells were recovered, centrifuged at 1500 rpm (453 ***g***) for 10 min and re-suspended in proliferation medium consisting of IMDM with Glutamax, 1% penicillin/streptomycin, 20% fetal calf serum and 1% chicken embryo extract. Once primary myoblasts had reached 60-70% confluence, cells were switched to differentiation medium consisting of IMDM with Glutamax, 1% penicillin/streptomycin and 2% horse serum (Gibco, 26050088) (Fig. 1, day −3). The day after (Fig. 1, day −2), myotubes were coated with Matrigel and kept in fresh differentiation medium for 48 h at 37°C in a 5% CO_2_ incubator before plating of spinal cord explants.

### Embryonic spinal cord explants isolation

Pregnant E13 Sprague-Dawley rats were euthanized with CO_2_ (Fig. 1, Day 0). Embryo chain was sampled in HBSS (Gibco, 14170) supplemented with 10% penicillin/streptomycin. Each embryo was isolated and rinsed in HBSS with penicillin/streptomycin solution and then decapitated. Spinal cords were isolated from the rest of the embryo using Student Vannas spring scissors (Fine Science Tools, 15001-08/15000-00) and Student Dumont forceps (Fine Science Tools, 11200-33/11297-10/11254-20) under binoculars. Blood vessels, muscles and connective tissue were removed delicately so that only the spinal cord with the dorsal root ganglia is left. The spinal cord was cut transversally in small explants with at least one dorsal root ganglion attached to the explant.

### Co-culture of neuronal cells and muscle fibers

Differentiation medium was removed from muscle myotubes (Fig. 1, Day 0). Spinal cord explants were plated very delicately on myotubes (four or five explants per dish) and supplemented with 100 µl of co-culture medium consisting of DMEM with Glutamax (Gibco, 61965-026), 25% of medium 199 with Glutamax (Gibco, 41150-020), 5% fetal bovine serum, 1% penicillin/streptomycin, 20 µg/ml insulin, 10 ng/ml GDNF, 10 ng/ml BDNF, 10 ng/ml CNTF. Co-culture medium was added dropwise directly to avoid detachment of the explants. Co-cultures were left at 37°C in 5% CO_2_ for 3-4 h to allow adhesion of explants. Co-culture medium was added very delicately up to 600 µl. The following day 400 µl of co-culture medium was added to the co-culture. At day 3 post-co-culturing, a layer of Matrigel was used to coat the co-culture in order to provide a three-dimensional environment to the culture and keep structures tightly bound. Medium was changed every two/three days accordingly to cell state. TTX was added at day 12 post-co-culturing (final concentration 1 µM, Alomone Labs) (Fig. 1). Co-cultures were fixed at day 14 post-co-culturing.

### Fixation and immunocytochemistry

Co-cultures were washed twice with PBS (Gibco ref 14190-094). Fixation was done in either 4% paraformaldehyde (Electron Microscopy Science, 15710-S) for 20 min at room temperature (RT) or in acetone/methanol solution (ratio 1:1) for 6 min at −20°C according to the specific requirements of each antibody. Alpha-bungarotoxin (BTX) labeling of AChRs was performed with 5 µg/ml TRITC-BTX (Sigma-Aldrich, T0195) in PBS for 15 min at RT prior to permeabilization and prior to acetone/methanol fixation. After BTX staining, cells were washed twice with PBS. Permeabilization was performed in PBS 5% Triton X-100 (Sigma-Aldrich, X100) for 5 min at RT. Cells were washed twice in PBS and then saturated in PBS containing 5% bovine serum albumin (BSA) and 10% goat serum (Gibco, 16210-064) for 1 h at RT. Primary antibodies were incubated overnight at 4°C in PBS containing 5% BSA and 0.1% saponin. Co-cultures were washed 3×10 min with PBS at RT under slow agitation and stained with the corresponding secondary antibodies supplemented with DAPI for 1 h at RT. Co-cultures were washed 3×10 min with PBS at RT and then mounted in Fluoromount medium (Fluromount-G, Southern Biotech, 0100-01) and analyzed using confocal microscopy (Leica SPE confocal microscope with a 63×1.3 NA Apo objective).

### Primary antibodies

The following antibodies were used: mouse anti-βIII tubulin (R&D systems, MAB1195, clone #TuJ1, 1/400), rabbit anti-ryanodine receptor (Braubach et al., 2014) (Millipore, AB9078, 1/200), goat anti-VaChT (Atasoy et al., 2014) (Millipore, ABN100, 1/100), goat anti-ChAT (Sümbül et al., 2014) (Millipore, AB144, 1/100), chicken anti-neurofilament H (NFH) (Wainger et al., 2015) (Millipore, AB5539, 1/400), mouse anti-dihydropyridine receptor (DHPR) (Bradley et al., 2014) (Abcam, Ab2864, 1/400), mouse anti-synaptotagmin (Wong et al., 2014) (Abcam, ab13259 clone ASV30, 1/100), mouse anti-α-actinin (Falcone et al., 2014) (Sigma-Aldrich, A5044 clone BM-75.2, 1/500), mouse anti-sodium channel pan (Bailey et al., 2003) (Sigma-Aldrich, clone K58/35, S8809, 1/100), mouse anti-ankyrin (Bailey et al., 2003) (Thermo Scientific, 33-8800, clone 4G3F8, 1/100), mouse anti-bassoon (Jing et al., 2013) (Abcam, ab82958, 1/100), rabbit anti-glial fibrillary acidic protein (GFAP) (Achtstätter et al., 1986) (Dako, Z0334, 1/100), mouse anti-oligodendrocytic marker O4 (Paintlia et al., 2004) (Sigma-Aldrich, O7139, clone O4, 1/400), rabbit anti-MuSK (serum T194, gift from Markus Ruegg, Biozentrum, University of Basel, Switzerland, 1/500), mouse anti-rapsyn [Abcam, ab11423 (1234), 1/200], mouse anti-Syne1 (clone 8c3, gift from Glenn Morris, Keele University, UK, 1/200).

### Quantification of peripheral nuclei

Myofibers were stained for DHPR and DAPI and images were acquired with a Leica SPE confocal using a 40×1.15 NA ACS Apo objective at different *z* positions. Nuclei extruding the myofiber periphery were considered to be peripheral. A minimum of 20 fibers were counted per condition in three independent experiments.

### Quantification of transversal triads

Myofibers were stained for DHPR and DAPI and images were acquired with a Leica SPE confocal 40×1.15 NA ACS Apo objective. Fibers with more than 50% transversal triads were scored as positive. A minimum of 20 fibers were counted per condition in three independent experiments.

### Fiber thickness quantification

Myofibers were stained for RyR and images were acquired with a Leica SPE confocal 40×1.15 NA ACS Apo objective. Average of three measurements per fiber was calculated for fiber thickness. A minimum of 20 fibers were counted per condition in three independent experiments.

### Fluorescence and live imaging

Epi-fluorescence images were acquired using a Nikon Timicroscope equipped with a CoolSNAP HQ2 camera (Roper Scientific), an *xy*-motorized stage (Nikon) driven by Metamorph (Molecular Devices), and 4×0.13 NA, 10×0.30 NA and 20× 0.45 NA PlanApo oil immersion objectives. Confocal images were acquired using Leica SPE confocal microscope with a 40×1.15 NA ACS Apo objective. Live imaging was performed in an insulated temperature controlled chamber (Okolab) to maintain cultures at 37°C and 5% CO_2_ (Okolab) using a 20×0.3 NA PL Fluo dry objective.

### Image analysis

Images were analyzed using ImageJ software (imagej.nih.gov/ij/). Images of *z*-projections are specified in corresponding figure legends. 3D rendering (Fig. 5B′) was performed using Voxx 2.1 software (Indiana University).

### Tetrodotoxin experiment

For action potential-blocking experiments, the selective Na_V_ channels blocker tetrodotoxin (final concentration 1 µM, Alomone Labs) was applied to the co-culture medium. Response in myofibers was recorded by video microscopy at 2 frames per second and analyzed using Metamorph 7.1 (Molecular Devices). Contractions of myofibers were monitored by following displacement of the plasma membrane of one point over time using the Track points plug in.

### Fluorescence *in situ* hybridization

For RNA FISH, we modified the protocol provided by Stellaris (Biosearch Technologies CA, USA). Briefly, cells were first incubated with bungarotoxin (5 µg/ml) conjugated with a 488 nm fluorophore and washed twice with PBS prior to fixation with 4% formaldehyde and permeabilization with 70% ethanol for 10 min at 4°C. Probes conjugated with 570 nm fluorophore against AChRε (Stellaris) were incubated at 125 nM in 100 mg/ml dextran sulfate and 10% formamide in 2× SSC with cells for 16 h at 37°C. After washing with 10% formamide in 2× SSC, cells were incubated with primary antibody against Tuj1, followed by incubation with secondary antibody and DAPI at 5 ng/ml. After washes with 10% formamide in 2× SSC, cells were covered by Vectashield and directly imaged using a wide-field fluorescence microscope.

### Electrophysiology recordings

For technical purposes, electrophysiological studies were performed in conditions of diluted Matrigel. These changes lead to decrease myofiber differentiation, but we postulated that if in these conditions the NMJs were functional, it would allow us to demonstrate functionality in the usual co-culture conditions. Intracellular recordings were performed at room temperature in a solution containing (in mM): 145 NaCl, 3 KCl, 2 CaCl_2_, 1 MgCl_2_, 10 HEPES (pH 7.4) and 11 glucose. Sharp pipettes were made from borosilicate glass (Clark Electromedical Instruments, Reading, England), pulled on a P-1000 puller (Sutter Instrument Company, Novato, CA, USA) and had a resistance of 40-60 MΩ when filled with a 3 M KCl solution. Membrane potential was recorded using a SEC-0.5X amplifier (npi electronic, Tamm, Germany) and digitized by a 16 bit A/D converter (Digidata 1322A, Axon Instruments, Union City, CA, USA).

### Statistical analysis

Statistical significance was determined using GraphPad Prism (GraphPad Prism Software version 6). Statistical tests used have been mentioned in the corresponding figure legends. *P*≤0.05 was considered as significant.
